# Proteomes: A New Proteomic Journal

**DOI:** 10.3390/proteomes1010001

**Published:** 2012-10-26

**Authors:** Jacek R. Wiśniewski

**Affiliations:** Department of Proteomics and Signal Transduction, Max-Planck-Institute for Biochemistry, Am Klopferspitz 18, D-82152 Martinsried, Germany; Email: jwisniew@biochem.mpg.de; Tel.: +49-89-85-78-2205; Fax: +49-89-85-78-2219

In the early years of proteomics, mass spectrometry served only as a technique in protein chemistry facilitating the characterization of purified proteins and mapping their posttranslational modifications (PTMs). A bit later this technique almost completely replaced Edman degradation and amino acid analysis. The continuous development of the mass spectrometry techniques created a huge analytical potential allowing the study of nearly complete proteomes in single experiments. This evolution distanced proteomics from protein chemistry and placed it in a novel position. Its capability to identify and quantify in parallel thousands of proteins and their modifications at minute sample amount requirements is one of the most fascinating technological advances in biology today. 

There are several major areas of proteomics, such as mapping of proteomes, identification of posttranslational modifications and discovering interactions. Notably, proteomics has not only the capability to unveil protein composition but is also suitable for studying protein levels and their changes on a system wide scale. Currently, we can map more than 10,000 proteins in a single cell type and estimate the concentration of each protein. Even though some estimates are rough, they place proteomics closer to biochemistry and are helpful in better understanding a variety of phenomena in physiology, growth control and signaling. 

Identification of posttranslational modification is one of the most attractive areas of proteomics. Expensive and laborious tasks that have been necessary for mapping PTMs using classical biochemistry, including labeling with radioactive isotopes, purification of peptides from isolated proteins purifications, and sequencing, were substituted by mass spectrometry based proteomics. Thanks to these developments, analysis of PTMs has become very popular, in particular because large numbers of modification sites can be identified in a relatively simple way by analyzing enriched fractions or just within complete cell lysates. In-depth proteomic studies allow mapping of thousands of phosphorylation, acetylation, or glycosylation sites occurring in a single population of cells or in a tissue, and position proteomics in a key role for studying modifications in a biological system’s manner. Supplementing the identification with protein abundance and site-occupancy information will lead to validation of thermodynamic significance of individual modifications. 

Analysis of clinical samples is another popular area of proteomics. Its landscape is shared by the identification of novel biomarkers and discovery of potential drug targets. The major challenges in this area are the limited availability of quality samples and the abundance of housekeeping proteins such as albumin in plasma, or titin in muscle tissue. The presence of such proteins impedes identification of lower abundant molecules that are the focus of analyses. Development of approaches to bypass these difficulties and to facilitate the discovery process is the aim of many researchers. 

Although proteomic technologies were frequently understood as something ‘under construction’ doubtless proteomic technologies are now ready to provide novel insights in biological processes. We launch *Proteomes* with the certainty that we can attract high quality manuscripts which will cover various fields of proteomics. We expect that *Proteomes* will publish articles in which proteomic technologies will play an essential role in solving questions and providing new insights in biology, biochemistry, and molecular biology.

